# Supplementary material for: DINGO: increasing the power of locus discovery in maternal and fetal genome-wide association studies of perinatal traits
Source: Nat Commun. 2024 Oct 26;15:9255. doi: 10.1038/s41467-024-53495-9 (PMC11513127; doi:10.1038/s41467-024-53495-9)
Supplement: Supplementary file 1 — Supplementary Information [file 41467_2024_53495_MOESM1_ESM.pdf]

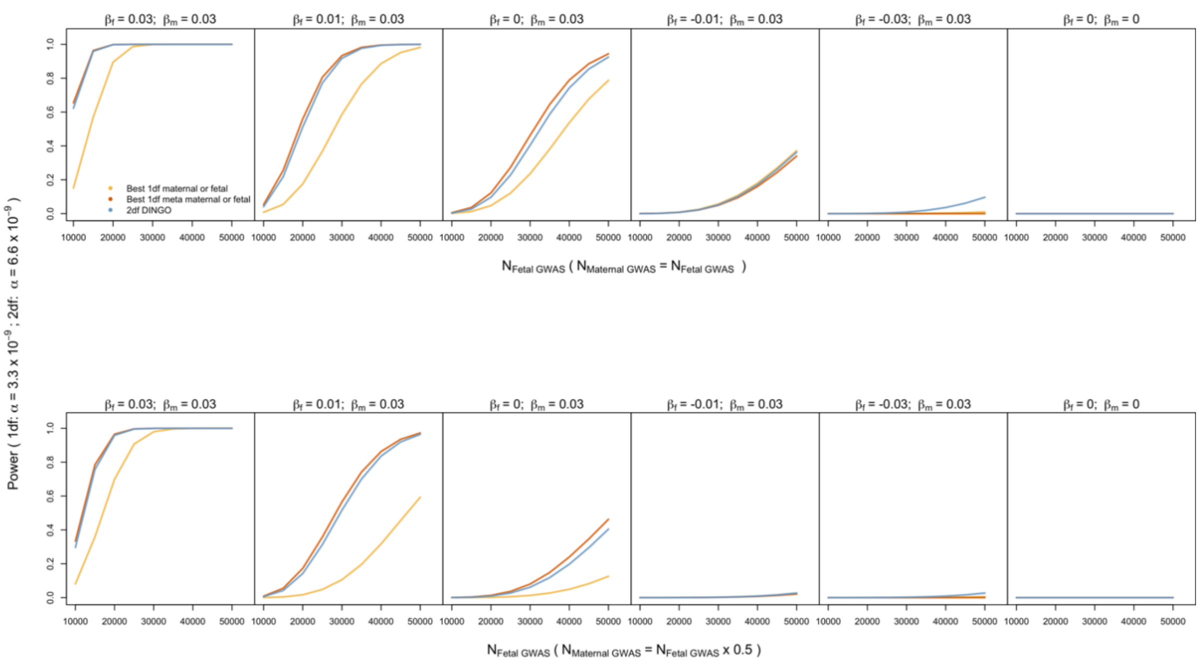

Supplementary Figure 1. Power to detect association (evaluated by asymptotic calculation) using a traditional strategy of analysing separate maternal and fetal GWASs, a strategy involving one-degree-of-freedom meta-analyses, and a strategy of performing two-degree-of-freedom T_{2df} tests across the genome. The effect of varying the sample size of the maternal GWAS is shown. β_m and β_f refer to maternal and fetal genetic effects on a standardized trait. No sample overlap. In the top row, the sample sizes for the maternal GWAS and fetal GWAS are the same. In the bottom row, the sample size for the maternal GWAS is half the fetal GWAS. For the traditional strategy of running separate maternal and fetal GWASs and the one-degree-of-freedom meta-analysis strategy, we set the alpha value to $\alpha = 3.3 \times 10^{-9}$ i.e. half the $\alpha = 6.6 \times 10^{-9}$ type I error rate of the two-degree-of-freedom T_{2df} test in order to take into account that we are performing twice the number of statistical tests in the former situations. In the case of the traditional strategy of running separate maternal and fetal GWASs and the one-degree-of-freedom meta-analysis strategy, we evaluated power with respect to whether *either* test met the criterion for genome-wide significance.

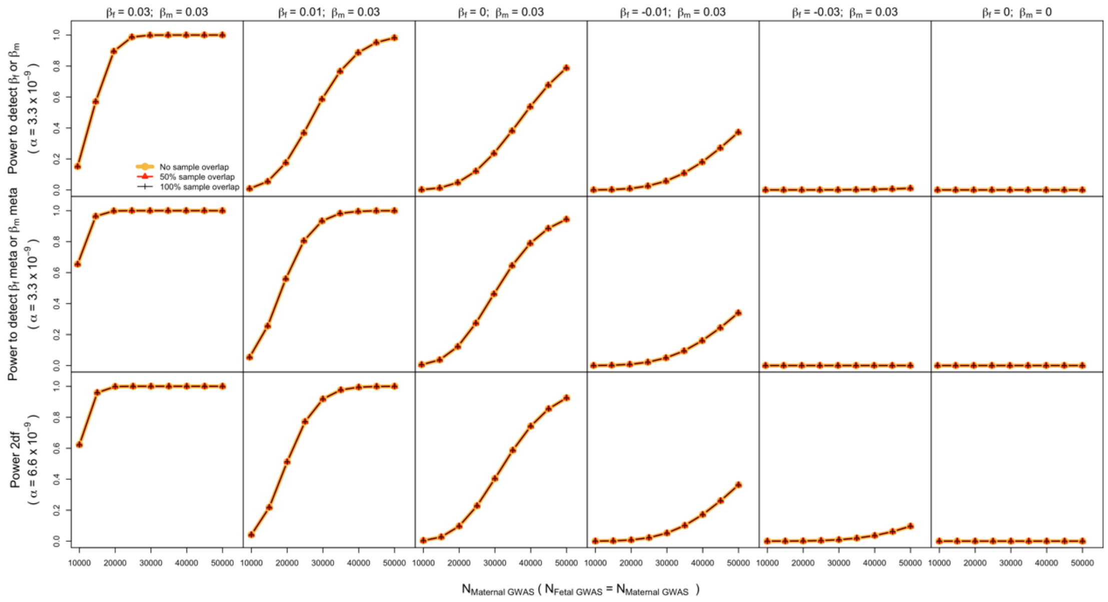

Supplementary Figure 2. Power to detect association (evaluated by asymptotic calculation) using a traditional strategy of analysing separate maternal and fetal GWASs (top panel), a strategy involving one-degree-of-freedom meta-analyses (middle panel), and a strategy of performing two-degree-of-freedom T_{2df} tests across the genome (bottom panel). The effect of varying the degree of sample overlap is shown. No residual correlation between maternal and offspring phenotypes (ρ = 0). β_m and β_f refer to maternal and fetal genetic effects on a standardized trait. For the traditional strategy of running separate maternal and fetal GWASs and the one-degree-of-freedom meta-analysis strategy, we set the alpha value to $\alpha = 3.3 \times 10^{-9}$ i.e. half the $\alpha = 6.6 \times 10^{-9}$ type I error rate of the two-degree-of-freedom T_{2df} test in order to take into account that we are performing twice the number of statistical tests in the former situations. In the case of the traditional strategy of running separate maternal and fetal GWASs and the one-degree-of-freedom meta-analysis strategy, we evaluated power with respect to whether *either* test met the criterion for genome-wide significance.

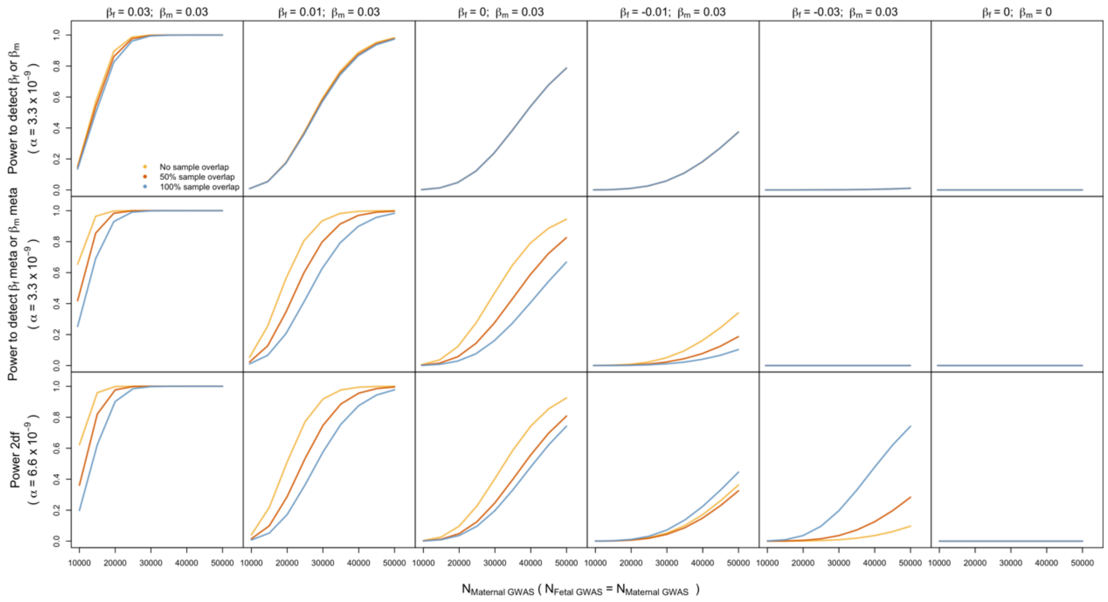

Supplementary Figure 3. Power to detect association (evaluated by asymptotic calculation) using a traditional strategy of analysing separate maternal and fetal GWASs (top panel), a strategy involving one-degree-of-freedom meta-analyses (middle panel), and a strategy of performing two-degree-of-freedom T_{2df} tests across the genome (bottom panel). The effect of varying the degree of sample overlap is shown. Scenarios shown involve high residual correlation between maternal and offspring phenotypes (ρ = 0.5). β_m and β_f refer to maternal and fetal genetic effects on a standardized trait. For the traditional strategy of running separate maternal and fetal GWASs and the one-degree-of-freedom meta-analysis strategy, we set the alpha value to $\alpha = 3.3\text{x}10^{-9}$ i.e. half the $\alpha = 6.6\text{x}10^{-9}$ type I error rate of the two-degree-of-freedom T_{2df} test in order to take into account that we are performing twice the number of statistical tests in the former situations. In the case of the traditional strategy of running separate maternal and fetal GWASs and the one-degree-of-freedom meta-analysis strategy, we evaluated power with respect to whether *either* test met the criterion for genome-wide significance.

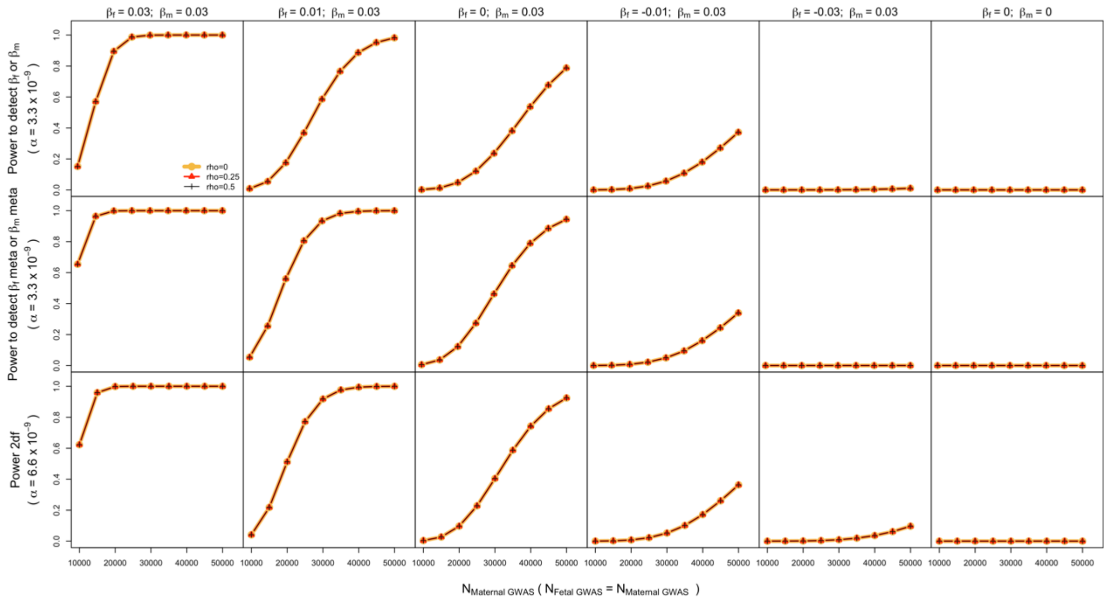

Supplementary Figure 4. Power to detect association (evaluated by asymptotic calculation) using a traditional strategy of analysing separate maternal and fetal GWASs (top panel), a strategy involving one-degree-of-freedom meta-analyses (middle panel), and a strategy of performing two-degree-of-freedom T_{2df} tests across the genome (bottom panel). The effect of varying the residual phenotypic correlation (ρ) is shown. No sample overlap between maternal and fetal GWASs. β_m and β_f refer to maternal and fetal genetic effects on a standardized trait. For the traditional strategy of running separate maternal and fetal GWASs and the one-degree-of-freedom meta-analysis strategy, we set the alpha value to $\alpha = 3.3 \times 10^{-9}$ i.e. half the $\alpha = 6.6 \times 10^{-9}$ type I error rate of the two-degree-of-freedom T_{2df} test in order to take into account that we are performing twice the number of statistical tests in the former situations. In the case of the traditional strategy of running separate maternal and fetal GWASs and the one-degree-of-freedom meta-analysis strategy, we evaluated power with respect to whether *either* test met the criterion for genome-wide significance.

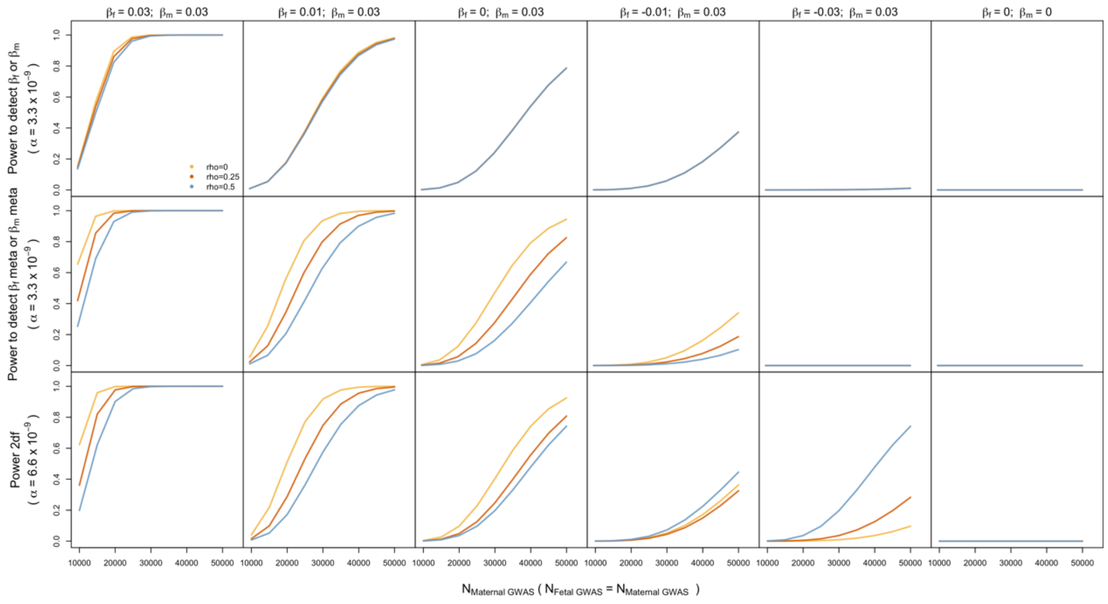

Supplementary Figure 5. Power to detect association (evaluated by asymptotic calculation) using a traditional strategy of analysing separate maternal and fetal GWASs (top panel), a strategy involving one-degree-of-freedom meta-analyses (middle panel), and a strategy of performing two-degree-of-freedom T_{2df} tests across the genome (bottom panel). The effect of varying the residual phenotypic correlation (ρ) is shown. 100% sample overlap between maternal and fetal GWASs. β_m and β_f refer to maternal and fetal genetic effects on a standardized trait. For the traditional strategy of running separate maternal and fetal GWASs and the one-degree-of-freedom meta-analysis strategy, we set the alpha value to $\alpha = 3.3 \times 10^{-9}$ i.e. half the $\alpha = 6.6 \times 10^{-9}$ type I error rate of the two-degree-of-freedom T_{2df} test in order to take into account that we are performing twice the number of statistical tests in the former situations. In the case of the traditional strategy of running separate maternal and fetal GWASs and the one-degree-of-freedom meta-analysis strategy, we evaluated power with respect to whether *either* test met the criterion for genome-wide significance.

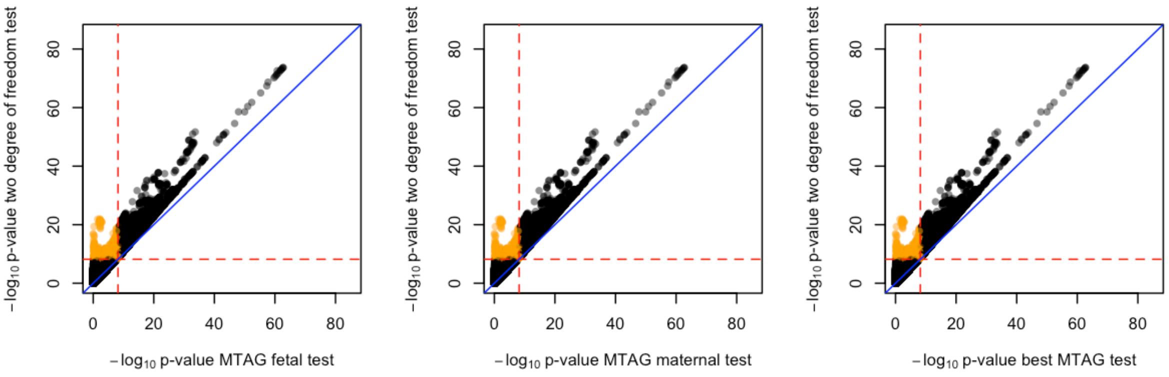

Supplementary Figure 6. P-value scatter plots comparing MTAG results versus the two-degree-of-freedom test in the GWAS analysis of birth weight (n = 423,683) and offspring birth weight (n = 270,002). The $-\log_{10}$p-value from the two-degree-of-freedom test was compared against the $-\log_{10}$p-value of SNPs from (left) the MTAG results for own birth weight, (middle) the MTAG results for offspring birth weight, and (right) the strongest MTAG p-value at the locus. Red dashed lines denote the genome-wide significant thresholds of $\alpha = 3.3 \times 10^{-9}$ for the one-degree-of-freedom MTAG analyses and $\alpha = 6.6 \times 10^{-9}$ for the two-degree-of-freedom test. Blue diagonal lines indicate x=y. Orange circles are SNPs that are only significant using the two-degree-of-freedom test.

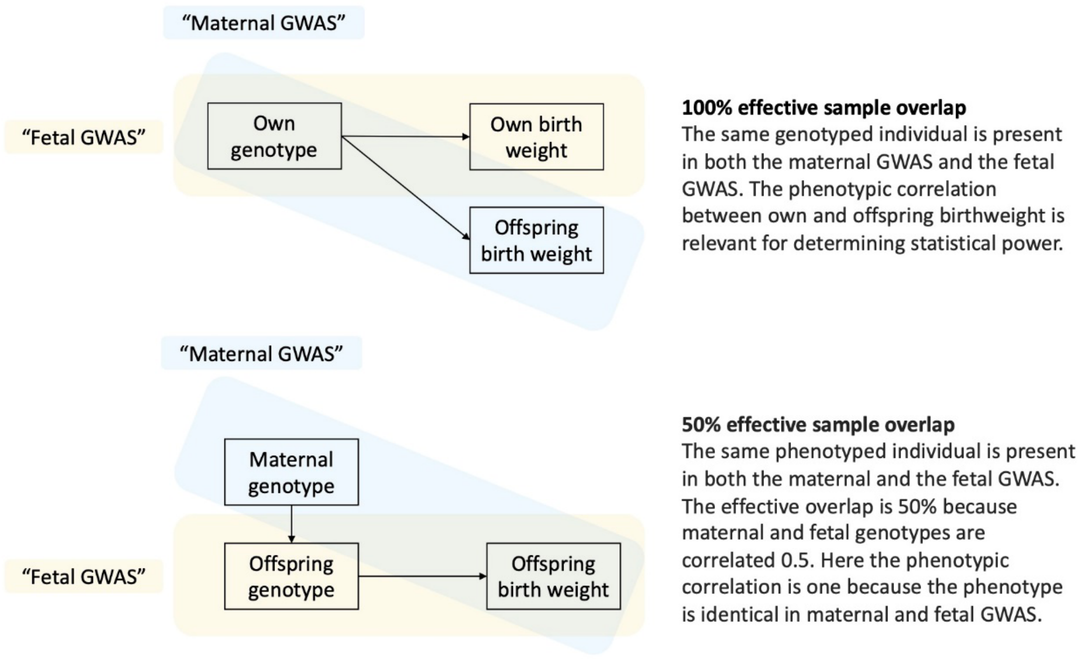

Supplementary Figure 7. Effective number of individuals in maternal and fetal GWAS. The top part of the figure illustrates a single genotyped individual who is present in both the maternal (i.e. GWAS of offspring birth weight) and fetal GWAS (i.e. GWAS of own birth weight). The UK Biobank is an example of this sort of situation, where genotyped females self-report their own birth weight and the birth weight of their first offspring. Because the same individuals are present in both GWAS, the effective sample overlap is 100%, however the phenotypes differ (i.e. own birthweight versus offspring birthweight). The correlation between the phenotypes affects statistical power. The bottom part of the figure displays a genotyped mother-offspring pair, where only offspring birth weight is measured. Here the effective sample overlap is 50%, because it is not the same genotyped individual in the maternal and fetal GWAS, but rather first-degree relatives (who are genetically correlated 0.5). The phenotypic correlation is one because the phenotype is identical in maternal and fetal GWAS.

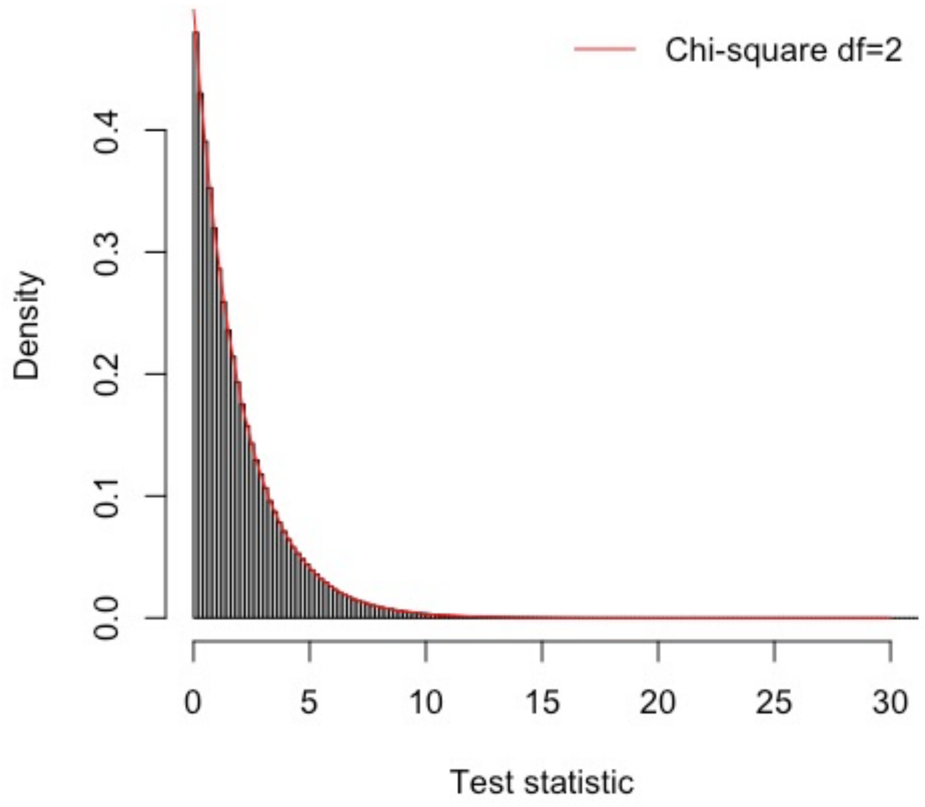

Supplementary Figure 8. Empirical distribution of the two-degree-of-freedom test statistic T_{2df} across the genome under a single permuted dataset in the UK Biobank. Summary results statistics were thinned by extracting one in every thousand SNPs (i.e. to correct for dependencies that might arise through linkage disequilibrium at close by markers) and checked against known distributions using the one-sample Kolmogorov-Smirnov test. The red line indicates the expected probability density for a two-degree-of-freedom chi-square distribution. The empirical distribution closely matched the two-degree-of-freedom chi-square distribution (Kolmogorov-Smirnov p-value = 0.86).

Supplementary Note

For the derivations below, we assume that the population model for the phenotype is given by the linear function:

$$y_i = \alpha + \beta_f x_{f_i} + \beta_m x_{m_i} + u_i$$

where y is the phenotype to be modelled, x_f and x_m are the dosage of an individual's own (i.e. fetal) and their mother's SNP (i.e. maternal) respectively, β_f and β_m are the true population fetal and maternal genetic effects on the phenotype at these SNPs, α is an intercept, u is an error term that is mean independent of the SNP dosages i.e. $\mathrm{E}\left(u|x_f, x_m\right) = 0$ and represents the sum total of all unmodelled influences on y, and the *i* subscript indexes individual *i*.

Regression coefficients from maternal and fetal GWASs are biased and inconsistent in the presence of both maternal and fetal genetic effects at the same locus

The simple regression of an individual's own phenotype (y) on their own SNP dosage (x_f) is an inconsistent estimator of the true direct fetal genetic effect (β_f) in the presence of maternal genetic effects (β_m) at the same locus i.e.:

$$\mathrm{plim}(\hat{b}_f) = \mathrm{plim}\left(\frac{\mathrm{cov}(y,x_f)}{\mathrm{var}(x_f)}\right) = \frac{\mathrm{COV}(y,x_f)}{\mathrm{VAR}(x_f)} = \frac{\mathrm{VAR}(x_f)\left(\beta_f + \frac{1}{2}\beta_m\right)}{\mathrm{VAR}(x_f)} = \beta_f + \frac{1}{2}\beta_m$$

where cov and var refers to the sample covariance and variance, COV and VAR the population variance and covariance, and plim is the probability limit.

Likewise, the simple regression of an individual's own phenotype (y_i) on their own SNP dosage (x_{f_i}) is a biased estimator of the true direct fetal genetic effect (β_f) in the presence of indirect maternal genetic effects (β_m) at the same locus i.e.:

$$\mathrm{E}(\hat{b}_f) = \mathrm{E}(\mathrm{E}(\hat{\beta}_{f_{OLS}}|\boldsymbol{x_f}))$$

$$= \mathrm{E}(\mathrm{E}(\frac{\sum_{i=1}^{N}\left(x_{f_i} - \bar{x}_f\right)y_i}{\sum_{i=1}^{N}\left(x_{f_i} - \bar{x}_f\right)x_{f_i}}|\boldsymbol{x_f}))$$

$$= \mathrm{E}(\mathrm{E}(\frac{\sum_{i=1}^{N}\left(x_{f_i} - \bar{x}_f\right)\left(\alpha + \beta_f x_{f_i} + \beta_m x_{m_i} + u_i\right)}{\sum_{i=1}^{N}\left(x_{f_i} - \bar{x}_f\right)x_{f_i}}|\boldsymbol{x_f}))$$

$$= \mathrm{E}(\mathrm{E}(\frac{\alpha\sum_{i=1}^{N}\left(x_{f_i} - \bar{x}_f\right)}{\sum_{i=1}^{N}\left(x_{f_i} - \bar{x}_f\right)x_{f_i}} + \frac{\beta_f\sum_{i=1}^{N}\left(x_{f_i} - \bar{x}_f\right)x_{f_i}}{\sum_{i=1}^{N}\left(x_{f_i} - \bar{x}_f\right)x_{f_i}} + \frac{\beta_m\sum_{i=1}^{N}\left(x_{f_i} - \bar{x}_f\right)x_{m_i}}{\sum_{i=1}^{N}\left(x_{f_i} - \bar{x}_f\right)x_{f_i}} + \frac{\sum_{i=1}^{N}\left(x_{f_i} - \bar{x}_f\right)u_i}{\sum_{i=1}^{N}\left(x_{f_i} - \bar{x}_f\right)x_{f_i}}|\boldsymbol{x_f}))$$

$$= 0 + \beta_f + \beta_m\frac{\mathrm{cov}(x_f, x_m)}{\mathrm{var}(x_f)} + \frac{\sum_{i=1}^{N}\left(x_{f_i} - \bar{x}_f\right)}{\sum_{i=1}^{N}\left(x_{f_i} - \bar{x}_f\right)x_{f_i}}\mathrm{E}(\mathrm{E}(\,u_i|\boldsymbol{x_f}))$$

$$= \beta_f + \beta_m\frac{\mathrm{cov}(x_f, x_m)}{\mathrm{var}(x_f)}$$

$$= \beta_f + \frac{1}{2}\beta_m \text{ in large samples.}$$

By a similar process it can be shown that the simple regression of offspring phenotype on maternal SNP is a biased and inconsistent estimate of the true indirect maternal genetic effect (β_m) in the presence of direct fetal effects (β_f) at the same locus i.e.:

$$\text{plim}(\hat{b}_m) = \beta_m + \frac{1}{2}\beta_f$$

$$\text{E}(\hat{b}_m) = \beta_m + \beta_f \frac{\text{cov}(x_f, x_m)}{\text{var}(x_m)} = \beta_m + \frac{1}{2}\beta_f \text{ in large samples.}$$

Asymptotic distribution of the two-degree-of-freedom test T_{2df} under the null and alternative hypotheses

The joint sampling distribution of maternal and fetal effect estimates is bivariate normal in large samples:

$$\begin{pmatrix} \hat{\beta}_f \\ \hat{\beta}_m \end{pmatrix} \sim N\left(\begin{pmatrix} \beta_f \\ \beta_m \end{pmatrix}, \boldsymbol{\Sigma}\right)$$

$$\text{with } \boldsymbol{\Sigma} = \begin{pmatrix} \text{var}(\hat{\beta}_f) & \text{cov}(\hat{\beta}_f, \hat{\beta}_m) \\ \text{cov}(\hat{\beta}_f, \hat{\beta}_m) & \text{var}(\hat{\beta}_m) \end{pmatrix}$$

The T_{2df} test statistic is given by:

$$\text{T}_{2df} = \begin{pmatrix} \hat{\beta}_f & \hat{\beta}_m \end{pmatrix} \begin{pmatrix} \text{var}(\hat{\beta}_f) & \text{cov}(\hat{\beta}_f, \hat{\beta}_m) \\ \text{cov}(\hat{\beta}_f, \hat{\beta}_m) & \text{var}(\hat{\beta}_m) \end{pmatrix}^{-1} \begin{pmatrix} \hat{\beta}_f \\ \hat{\beta}_m \end{pmatrix} = \hat{\boldsymbol{\beta}}'\boldsymbol{\Sigma}^{-1}\hat{\boldsymbol{\beta}}$$

Let

$$\mathbf{S} = \begin{matrix} \text{SE}(\hat{\beta}_f) & 0 \\ 0 & \text{SE}(\hat{\beta}_m) \end{matrix}$$

We can therefore rewrite T_{2df} as:

$$\text{T}_{2df} = \hat{\boldsymbol{\beta}}'\mathbf{S}^{-1\prime}\mathbf{S}'\boldsymbol{\Sigma}^{-1}\mathbf{S}\mathbf{S}^{-1}\hat{\boldsymbol{\beta}} = \mathbf{z}'\mathbf{R}^{-1}\mathbf{z}$$

where $\mathbf{z} \sim \text{N}(\boldsymbol{\mu}, \mathbf{R})$ and $\mathbf{R}$ is the correlation matrix of fetal and maternal effect estimates.

Let $\boldsymbol{\Lambda}$ and $\mathbf{U}$ be the diagonal matrix of eigenvalues and the orthogonal matrix of eigenvectors of $\mathbf{R}$ respectively. Performing a eigen decomposition on $\mathbf{R}$ and inverting we have:

$$\mathbf{R}^{-1} = \mathbf{U}\boldsymbol{\Lambda}^{-1}\mathbf{U}'$$

If we let $\mathbf{w} = \boldsymbol{\Lambda}^{-\frac{1}{2}}\mathbf{U}'\mathbf{z}$, then

$$\text{T}_{2df} = \mathbf{z}'\mathbf{U}\boldsymbol{\Lambda}^{-1}\mathbf{U}'\mathbf{z} = (\mathbf{z}'\mathbf{U}\boldsymbol{\Lambda}^{-\frac{1}{2}})(\boldsymbol{\Lambda}^{-\frac{1}{2}}\mathbf{U}'\mathbf{z}) = \mathbf{w}'\mathbf{w}$$

with E($\mathbf{w}$) = $\boldsymbol{\Lambda}^{-\frac{1}{2}}\mathbf{U}'\boldsymbol{\mu}$ and Var($\mathbf{w}$) = $\boldsymbol{\Lambda}^{-\frac{1}{2}}\mathbf{U}'\mathbf{R}\mathbf{U}\boldsymbol{\Lambda}^{-\frac{1}{2}} = \boldsymbol{\Lambda}^{-\frac{1}{2}}\mathbf{U}'\mathbf{U}\boldsymbol{\Lambda}\mathbf{U}'\mathbf{U}\boldsymbol{\Lambda}^{-\frac{1}{2}} = \mathbf{I}$, where

$$\mathbf{w} \sim N(\boldsymbol{\Lambda}^{-\frac{1}{2}}\mathbf{U}'\boldsymbol{\mu}, \mathbf{I})$$

Under the null hypothesis of no maternal and fetal effect at the variant being tested, $\boldsymbol{\mu} = \mathbf{0}$. In this case, $\mathbf{w}$ is a 2 × 1 vector of standard normal variables. Thus, under H_0 the T_{2df} test statistic follows a central chi-squared distribution with two degrees of freedom:

$$T_{2df} \sim \chi_2^2$$

Under the alternative hypothesis that there exists a maternal and fetal effect of the SNP, $\boldsymbol{\mu} \neq \mathbf{0}$. Let $\boldsymbol{\eta} = \boldsymbol{\Lambda}^{-\frac{1}{2}}\mathbf{U}'\boldsymbol{\mu}$, then $\mathbf{w} \sim N(\boldsymbol{\eta}, \mathbf{I})$. The sum of squares of $\mathbf{w}$ follows a non-central chi-square distribution with two degrees of freedom and non-centrality parameter NCP = $\boldsymbol{\eta}'\boldsymbol{\eta} = \boldsymbol{\mu}'\mathbf{U}\boldsymbol{\Lambda}^{-\frac{1}{2}}\boldsymbol{\Lambda}^{-\frac{1}{2}}\mathbf{U}'\boldsymbol{\mu} = \boldsymbol{\mu}'\boldsymbol{\Sigma}^{-\mathbf{1}}\boldsymbol{\mu}$. It follows that under H_1:

$$T_{2df} \sim \chi_2^2(\boldsymbol{\mu}'\boldsymbol{\Sigma}^{-\mathbf{1}}\boldsymbol{\mu})$$

Distribution of the two-degree-of-freedom test statistic T_{2df} under the Null Hypothesis of no association between SNPs and trait

To validate whether our test statistic was distributed as a two-degree-of-freedom chi-square test under the Null Hypothesis of no association, we applied our model to a single permuted dataset in the UK Biobank. We extracted phenotypes of birth weight and offspring birth weight from the UK Biobank. Exclusion criteria included multiple births, inconsistent reports of birth weight between time points, birth weight >4.5 kilograms or <2.5 kilograms, withdrawal from the UK Biobank, and non-European ancestry. Only unrelated individuals, defined as having genomic similarity less than 3rd degree relatives[1], were included in the final sample. This resulted in 118,016 individuals with own birth weight only, 91,164 mothers with offspring birth weight only, and 103,701 mothers with both own and offspring birth weight. Own birth weight was regressed on sex and assessment centre (offspring birth weight was regressed on assessment centre only since sex was not available), and the residuals were then transformed into z-scores. See the Warrington et al. (2019)[2] paper for details of phenotype preparation. We then permuted individuals' phenotypes with respect to their genotypes (i.e. individual's own birth weight and offspring birth weight were permuted together when both measures were present). We performed a fetal GWAS on own birth weight and a maternal GWAS on offspring birth weight using fastGWA[3]. We then applied the two-degree-of-freedom test to the regression coefficients and their standard errors from the two GWASs for each SNP across the genome. Given that mothers with own and offspring birth weight were included in both GWASs, we used LD score regression to estimate the degree of sample overlap (LD score intercept = 0.1192). Lastly, we thinned the summary results statistics by extracting one in every thousand results (i.e. to correct for dependencies that might arise through linkage disequilibrium at close by markers) checked the distribution of test statistics using the one-sample Kolmogorov-Smirnov test.

The empirical distribution of the test statistics under the Null Hypothesis of no association are shown in Supplementary Figure 1 and closely match a two-degree-of-freedom chi-square distribution (Kolmogorov-Smirnov p-value = 0.86).

The UK Biobank study was approved by the UK National Health Service National Research Ethics Service. Written consent was obtained from both the participants and their parents (for subjects younger than 18 years old). This study was approved by the Human Research Ethics Committee at the University of Queensland (approval number: 2019002705).

Derivation of the three-degree-of-freedom test

We assume that the population model for the phenotype is given by the linear function:

$$y = \alpha + \beta_f x_f + \beta_m x_m + \beta_p x_p + u$$

where y is the phenotype to be modelled, x_f , x_m and x_p are the dosage of fetal, maternal and paternal SNPs respectively, β_f β_m and β_p are the true population fetal, maternal and paternal genetic effects on the phenotype at these SNPs, α is an intercept, and u is an error term that is mean independent of the SNP dosages i.e. $E\left(u|x_f, x_m, x_p\right) = 0$ and represents the sum total of all unmodelled influences on y.

We can estimate the true direct fetal (β_f) and indirect maternal (β_m) and paternal (β_p) genetic effects using the following weighted linear combination of regression coefficients derived from empirical linear regressions of phenotype on fetal SNP ($\hat{b}_f$), maternal SNP ($\hat{b}_m$) and paternal SNP ($\hat{b}_p$):

$$\hat{\beta}_f = 2\hat{b}_f - \hat{b}_m - \hat{b}_p$$

$$\hat{\beta}_m = \frac{3}{2}\hat{b}_m - \hat{b}_f + \frac{1}{2}\hat{b}_p$$

$$\hat{\beta}_p = \frac{3}{2}\hat{b}_p - \hat{b}_f + \frac{1}{2}\hat{b}_m$$

and their standard errors:

$$\mathrm{SE}(\hat{\beta}_f) = \sqrt{4\mathrm{var}(\hat{b}_f) + \mathrm{var}(\hat{b}_m) + \mathrm{var}(\hat{b}_p) + 2\mathrm{cov}(\hat{b}_m, \hat{b}_p) - 4\mathrm{cov}(\hat{b}_f, \hat{b}_m) - 4\mathrm{cov}(\hat{b}_f, \hat{b}_p)}$$

$$= \sqrt{4\mathrm{var}(\hat{b}_f) + \mathrm{var}(\hat{b}_m) + \mathrm{var}(\hat{b}_p) + 2 \times \mathrm{int}_{m,p} \times \mathrm{SE}(\hat{b}_m)\mathrm{SE}(\hat{b}_p) - 4 \times \mathrm{int}_{f,m} \times \mathrm{SE}(\hat{b}_f)\mathrm{SE}(\hat{b}_m) - 4 \times \mathrm{int}_{f,p} \times \mathrm{SE}(\hat{b}_f)\mathrm{SE}(\hat{b}_p)}$$

$$\mathrm{SE}(\hat{\beta}_m) = \sqrt{\frac{9}{4}\mathrm{var}(\hat{b}_m) + \mathrm{var}(\hat{b}_f) + \frac{1}{4}\mathrm{var}(\hat{b}_p) - \mathrm{cov}(\hat{b}_f, \hat{b}_p) - 3\mathrm{cov}(\hat{b}_f, \hat{b}_m) + \frac{3}{2}\mathrm{cov}(\hat{b}_m, \hat{b}_p)}$$

$$= \sqrt{\frac{9}{4}\mathrm{var}(\hat{b}_m) + \mathrm{var}(\hat{b}_f) + \frac{1}{4}\mathrm{var}(\hat{b}_p) - \mathrm{int}_{f,p} \times \mathrm{SE}(\hat{b}_f)\mathrm{SE}(\hat{b}_p) - 3 \times \mathrm{int}_{f,m} \times \mathrm{SE}(\hat{b}_f)\mathrm{SE}(\hat{b}_m) + \frac{3}{2} \times \mathrm{int}_{m,p} \times \mathrm{SE}(\hat{b}_m)\mathrm{SE}(\hat{b}_p)}$$

$$\mathrm{SE}(\hat{\beta}_p) = \sqrt{\frac{9}{4}\mathrm{var}(\hat{b}_p) + \mathrm{var}(\hat{b}_f) + \frac{1}{4}\mathrm{var}(\hat{b}_m) - \mathrm{cov}(\hat{b}_f, \hat{b}_m) - 3\mathrm{cov}(\hat{b}_f, \hat{b}_p) + \frac{3}{2}\mathrm{cov}(\hat{b}_m, \hat{b}_p)}$$

$$= \sqrt{\frac{9}{4}\mathrm{var}(\hat{b}_p) + \mathrm{var}(\hat{b}_f) + \frac{1}{4}\mathrm{var}(\hat{b}_m) - \mathrm{int}_{f,m} \times \mathrm{SE}(\hat{b}_f)\mathrm{SE}(\hat{b}_m) - 3 \times \mathrm{int}_{f,p} \times \mathrm{SE}(\hat{b}_f)\mathrm{SE}(\hat{b}_p) + \frac{3}{2} \times \mathrm{int}_{m,p} \times \mathrm{SE}(\hat{b}_m)\mathrm{SE}(\hat{b}_p)}$$

Likewise, the sampling covariance of fetal, maternal, and paternal estimates is given by:

$$\mathrm{cov}(\hat{\beta}_f, \hat{\beta}_m) = 4\mathrm{cov}(\hat{b}_f, \hat{b}_m) + 2\mathrm{cov}(\hat{b}_f, \hat{b}_p) - 2\mathrm{cov}(\hat{b}_m, \hat{b}_p) - 2\mathrm{var}(\hat{b}_f) - \frac{3}{2}\mathrm{var}(\hat{b}_m) - \frac{1}{2}\mathrm{var}(\hat{b}_p)$$

$$= 4 \times \mathrm{int}_{f,m} \times \mathrm{SE}(\hat{b}_f)\mathrm{SE}(\hat{b}_m) + 2 \times \mathrm{int}_{f,p} \times \mathrm{SE}(\hat{b}_f)\mathrm{SE}(\hat{b}_p) - 2 \times \mathrm{int}_{m,p} \times \mathrm{SE}(\hat{b}_m)\mathrm{SE}(\hat{b}_p) - 2\mathrm{var}(\hat{b}_f) - \frac{3}{2}\mathrm{var}(\hat{b}_m) - \frac{1}{2}\mathrm{var}(\hat{b}_p)$$

$$\mathrm{cov}(\hat{\beta}_f, \hat{\beta}_p) = 4\mathrm{cov}(\hat{b}_f, \hat{b}_p) + 2\mathrm{cov}(\hat{b}_f, \hat{b}_m) - 2\mathrm{cov}(\hat{b}_m, \hat{b}_p) - 2\mathrm{var}(\hat{b}_f) - \frac{3}{2}\mathrm{var}(\hat{b}_p) - \frac{1}{2}\mathrm{var}(\hat{b}_m)$$

$$= 4 \times \mathrm{int}_{f,p} \times \mathrm{SE}(\hat{b}_f)\mathrm{SE}(\hat{b}_p) + 2 \times \mathrm{int}_{f,m} \times \mathrm{SE}(\hat{b}_f)\mathrm{SE}(\hat{b}_m) - 2 \times \mathrm{int}_{m,p} \times \mathrm{SE}(\hat{b}_m)\mathrm{SE}(\hat{b}_p) - 2\mathrm{var}(\hat{b}_f) - \frac{3}{2}\mathrm{var}(\hat{b}_p) - \frac{1}{2}\mathrm{var}(\hat{b}_m)$$

$$\mathrm{cov}(\hat{\beta}_m, \hat{\beta}_p) = \mathrm{var}(\hat{b}_f) + \frac{3}{4}\mathrm{var}(\hat{b}_m) + \frac{3}{4}\mathrm{var}(\hat{b}_p) + \frac{10}{4}\mathrm{cov}(\hat{b}_m, \hat{b}_p) - 2\mathrm{cov}(\hat{b}_f, \hat{b}_m) - 2\mathrm{cov}(\hat{b}_f, \hat{b}_p)$$

$$= \mathrm{var}(\hat{b}_f) + \frac{3}{4}\mathrm{var}(\hat{b}_m) + \frac{3}{4}\mathrm{var}(\hat{b}_p) + \frac{10}{4} \times \mathrm{int}_{m,p} \times \mathrm{SE}(\hat{b}_m)\mathrm{SE}(\hat{b}_p) - 2 \times \mathrm{int}_{f,m} \times \mathrm{SE}(\hat{b}_f)\mathrm{SE}(\hat{b}_m) - 2 \times \mathrm{int}_{f,p} \times \mathrm{SE}(\hat{b}_f)\mathrm{SE}(\hat{b}_p)$$

where $\mathrm{int}_{f,m}$, $\mathrm{int}_{f,p}$ and $\mathrm{int}_{m,p}$ are the estimated intercepts from the bivariate LD score regression of the maternal and fetal GWASs, the paternal and fetal GWASs, and the maternal and paternal GWASs respectively.

The joint sampling distribution of fetal, maternal and paternal effects is trivariate normal in large samples i.e.:

$$\begin{pmatrix} \hat{\beta}_f \\ \hat{\beta}_m \\ \hat{\beta}_p \end{pmatrix} \sim N\left(\begin{pmatrix} \beta_f \\ \beta_m \\ \beta_p \end{pmatrix}, \mathbf{\Sigma}\right)$$

$$\text{with } \mathbf{\Sigma} = \begin{pmatrix} \mathrm{var}(\hat{\beta}_f) & \mathrm{cov}(\hat{\beta}_f, \hat{\beta}_m) & \mathrm{cov}(\hat{\beta}_f, \hat{\beta}_p) \\ \mathrm{cov}(\hat{\beta}_f, \hat{\beta}_m) & \mathrm{var}(\hat{\beta}_m) & \mathrm{cov}(\hat{\beta}_m, \hat{\beta}_p) \\ \mathrm{cov}(\hat{\beta}_f, \hat{\beta}_p) & \mathrm{cov}(\hat{\beta}_m, \hat{\beta}_p) & \mathrm{var}(\hat{\beta}_p) \end{pmatrix}$$

We propose a test that considers the joint distribution of the three effect estimates. Using similar logic to above, it follows that under the null hypothesis (H_0) of no association between trait and maternal, paternal and fetal genotype, the test statistic:

$$\begin{pmatrix} \hat{\beta}_f & \hat{\beta}_m & \hat{\beta}_p \end{pmatrix} \begin{pmatrix} \mathrm{var}(\hat{\beta}_f) & \mathrm{cov}(\hat{\beta}_f, \hat{\beta}_m) & \mathrm{cov}(\hat{\beta}_f, \hat{\beta}_p) \\ \mathrm{cov}(\hat{\beta}_f, \hat{\beta}_m) & \mathrm{var}(\hat{\beta}_m) & \mathrm{cov}(\hat{\beta}_m, \hat{\beta}_p) \\ \mathrm{cov}(\hat{\beta}_f, \hat{\beta}_p) & \mathrm{cov}(\hat{\beta}_m, \hat{\beta}_p) & \mathrm{var}(\hat{\beta}_p) \end{pmatrix}^{-1} \begin{pmatrix} \hat{\beta}_f \\ \hat{\beta}_m \\ \hat{\beta}_p \end{pmatrix} \sim X_3^2$$

is distributed as a central chi-square statistic with three degrees of freedom.

Formulation of One-degree-of-freedom Meta-analytic Tests when Fathers' GWAS Available

It is straight forward to extend the meta-analytic test for fetal effects to include information from a GWAS of fathers i.e.:

$$w_1 = \frac{1}{\mathrm{var}(\hat{b}_f)}$$

$$w_2 = \frac{1}{4\mathrm{var}(\hat{b}_m)}$$

$$w_3 = \frac{1}{4\mathrm{var}(\hat{b}_p)}$$

$$\hat{\beta}_{f\_meta} = \frac{w_1\hat{b}_f + 2w_2\hat{b}_m + 2w_3\hat{b}_p}{w_1 + w_2 + w_3}$$

$$\mathrm{SE}(\hat{\beta}_{f\_meta}) = \sqrt{\begin{aligned}&(\frac{w_1}{w_1+w_2+w_3})^2\mathrm{var}(\hat{b}_f) + 4(\frac{w_2}{w_1+w_2+w_3})^2\mathrm{var}(\hat{b}_m) + 4(\frac{w_3}{w_1+w_2+w_3})^2\mathrm{var}(\hat{b}_p) + \\ &4\mathrm{cov}(\hat{b}_f,\hat{b}_m)\frac{w_1w_2}{(w_1+w_2+w_3)^2} + 4\mathrm{cov}(\hat{b}_f,\hat{b}_p)\frac{w_1w_3}{(w_1+w_2+w_3)^2} + 8\mathrm{cov}(\hat{b}_m,\hat{b}_p)\frac{w_2w_3}{(w_1+w_2+w_3)^2}\end{aligned}}$$

where $\hat{b}_f$ is the coefficient from the regression of own phenotype on own genotype, $\hat{b}_m$ is the coefficient from the regression of offspring phenotype on maternal genotype, $\hat{b}_p$ is the coefficient from the regression of offspring phenotype on paternal genotype, and $\hat{\beta}_{f\_meta}$ the inverse variance weighted estimate of the fetal effect across all three scans. The effective sample overlap between the scans is estimated using bivariate LD score regression and the covariance between the regression coefficients estimated from this quantity:

$$\widehat{\mathrm{cov}}(\hat{b}_f,\hat{b}_m) \approx \frac{N_{S\_fm}}{\sqrt{N_fN_m}}\rho_{fm}\sqrt{\mathrm{var}(\hat{b}_f)\mathrm{var}(\hat{b}_m)}$$

$$= \widehat{int}_{fm} \times \mathrm{SE}(\hat{b}_f)\mathrm{SE}(\hat{b}_m)$$

$$\widehat{\mathrm{cov}}(\hat{b}_f,\hat{b}_p) \approx \frac{N_{S\_fp}}{\sqrt{N_fN_p}}\rho_{fp}\sqrt{\mathrm{var}(\hat{b}_f)\mathrm{var}(\hat{b}_p)}$$

$$= \widehat{int}_{fp} \times \mathrm{SE}(\hat{b}_f)\mathrm{SE}(\hat{b}_p)$$

$$\widehat{\mathrm{cov}}(\hat{b}_m,\hat{b}_p) \approx \frac{N_{S\_mp}}{\sqrt{N_mN_p}}\rho_{mp}\sqrt{\mathrm{var}(\hat{b}_m)\mathrm{var}(\hat{b}_p)}$$

$$= \widehat{int}_{mp} \times \mathrm{SE}(\hat{b}_m)\mathrm{SE}(\hat{b}_p)$$

where N_f is the sample size of the GWAS of own genotype and own outcome, N_m is the sample size of the GWAS of maternal genotype and offspring phenotype, N_p is the sample size of the GWAS of paternal genotype and offspring phenotype, N_{S_fm}, N_{S_fp}, and N_{S_mp} are the effective number of overlapping individuals across the relevant GWASs, and $\widehat{int}_{fm}$, $\widehat{int}_{fp}$, and $\widehat{int}_{mp}$ are the associated estimated bivariate LD score regression intercepts. The parameters ρ_{fm}, ρ_{fp}, and ρ_{mp} refers to the correlation between the phenotype in the relevant overlapping individuals.

We note that the effective sample overlap between maternal and paternal scans is likely to be small but could still be non-zero (e.g. for example if brothers and their sisters were in the fathers' and mothers' GWAS respectively).

Power of the one-degree-of-freedom unconditional fetal and maternal test of association

Without loss of generality, we assume that the SNP marker and the outcome have been standardized to unit variance. The sampling variance of the (biased) ordinary least squares estimate of the fetal genetic effect is:

$$\mathrm{var}(\hat{b}_f) = \frac{(1-(\beta_f + \frac{1}{2}\beta_m)^2)}{N_f}$$

and likewise for (biased) ordinary least squares estimate of the maternal genetic effect:

$$\mathrm{var}(\hat{b}_m) = \frac{(1 - (\beta_m + \frac{1}{2}\beta_f)^2)}{N_m}$$

where N_f and N_m are the number of individuals in the fetal and maternal GWASs respectively.

It follows that the non-centrality parameter (NCP) for the unconditional one-degree-of-freedom tests for fetal and maternal effects are given by:

$$NCP_{FETAL} = \frac{(\beta_f + \frac{1}{2}\beta_m)^2}{\mathrm{var}(\hat{b}_f)}$$

$$NCP_{MATERNAL} = \frac{(\beta_m + \frac{1}{2}\beta_f)^2}{\mathrm{var}(\hat{b}_m)}$$

Asymptotic power can then be calculated as the area under the curve of a non-central chi-square distribution to the right of the significance threshold of interest:

$$Power = \int_{X_\alpha^{\prime 2}(v,0)}^{\infty} dX^{\prime 2}(v, \mathrm{NCP})$$

where $X_\alpha^{\prime 2}(v, 0)$ is the quantile of the 100 * (1-α) percentage point of the central χ^2 distribution with v degrees of freedom (one in this case), and NCP is the non-centrality parameter.

In order to estimate the power that either the maternal or fetal effect is significant we sample standardized effect sizes under the alternative hypothesis from a bivariate normal distribution with mean vector:

$$\boldsymbol{\mu} = \begin{matrix} \dfrac{\beta_f + \frac{1}{2}\beta_m}{\sqrt{\mathrm{var}(\hat{b}_f)}} \\ \dfrac{\beta_m + \frac{1}{2}\beta_f}{\sqrt{\mathrm{var}(\hat{b}_m)}} \end{matrix}$$

and covariance matrix:

$$\boldsymbol{\Sigma} = \begin{matrix} 1 & \dfrac{N_S}{\sqrt{N_f N_m}}\rho\sqrt{\mathrm{var}(\hat{b}_f)\mathrm{var}(\hat{b}_m)} \\ \dfrac{N_S}{\sqrt{N_f N_m}}\rho\sqrt{\mathrm{var}(\hat{b}_f)\mathrm{var}(\hat{b}_m)} & 1 \end{matrix}$$

Power is calculated as the proportion of draws where the absolute fetal *or* maternal effect exceeds the relevant critical value from the univariate standard normal distribution.

Power of the one-degree-of-freedom meta-analytic test for fetal and maternal effects

Recall that the weights for the meta-analysis of fetal genetic effects are given by:

$$w_1 = \frac{1}{\mathrm{var}(\hat{b}_f)}$$

$$w_2 = \frac{1}{4\mathrm{var}(\hat{b}_m)}$$

leading to the weighted estimate:

$$\hat{\beta}_{f\_meta} = \frac{w_1\hat{b}_f + 2w_2\hat{b}_m}{w_1 + w_2}$$

and its standard error:

$$\mathrm{SE}(\hat{\beta}_{f\_meta}) = \sqrt{(\frac{w_1}{w_1 + w_2})^2\mathrm{var}(\hat{b}_f) + 4(\frac{w_2}{w_1 + w_2})^2\mathrm{var}(\hat{b}_m) + 4\mathrm{cov}(\hat{b}_f, \hat{b}_m)\frac{w_1 w_2}{(w_1 + w_2)^2}}$$

Recall that the expectation for the fetal and maternal regression coefficients are given by:

$\mathrm{E}(\hat{b}_f) = \beta_f + \beta_m \frac{\mathrm{cov}(x_f, x_m)}{\mathrm{var}(x_f)} = \beta_f + \frac{1}{2}\beta_m$ in large samples,

$\mathrm{E}(\hat{b}_m) = \beta_m + \beta_f \frac{\mathrm{cov}(x_f, x_m)}{\mathrm{var}(x_m)} = \beta_m + \frac{1}{2}\beta_f$ in large samples

It follows that the non-centrality parameter for the meta-analytic fetal test is given by:

$$NCP_{FETAL\_META} = \frac{(\frac{w_1(\beta_f + \frac{1}{2}\beta_m) + 2w_2(\beta_m + \frac{1}{2}\beta_f)}{w_1 + w_2})^2}{\mathrm{SE}(\hat{\beta}_{f\_meta})^2}$$

The weights for the meta-analysis of maternal genetic effects are given by:

$$w_3 = \frac{1}{4\mathrm{var}(\hat{b}_f)}$$

$$w_4 = \frac{1}{\mathrm{var}(\hat{b}_m)}$$

leading to the weighted estimate:

$$\hat{\beta}_{f\_meta} = \frac{2w_3\hat{b}_f + w_4\hat{b}_m}{w_3 + w_4}$$

and its standard error

$$\mathrm{SE}(\hat{\beta}_{m\_meta}) = \sqrt{(\frac{w_3}{w_3 + w_4})^2\mathrm{var}(\hat{b}_m) + 4(\frac{w_3}{w_3 + w_4})^2\mathrm{var}(\hat{b}_f) + 4\mathrm{cov}(\hat{b}_f, \hat{b}_m)\frac{w_3 w_4}{(w_3 + w_4)^2}}$$

It follows that the non-centrality parameter for the meta-analytic maternal test is given by:

$$NCP_{MATERNAL\_META} = \frac{\left(\frac{2w_3(\beta_f + \frac{1}{2}\beta_m) + w_4(\beta_m + \frac{1}{2}\beta_f)}{w_3 + w_4}\right)^2}{\mathrm{SE}(\hat{\beta}_{m\_meta})^2}$$

In order to estimate the power that either the maternal or fetal meta-analytic effect is significant we sample standardized effect sizes under the alternative hypothesis from a bivariate normal distribution with mean:

$$\boldsymbol{\mu} = \begin{matrix} \dfrac{\frac{w_1(\beta_f + \frac{1}{2}\beta_m) + 2w_2(\beta_m + \frac{1}{2}\beta_f)}{w_1 + w_2}}{\mathrm{SE}(\hat{\beta}_{f\_meta})} \\ \dfrac{\frac{2w_3(\beta_f + \frac{1}{2}\beta_m) + w_4(\beta_m + \frac{1}{2}\beta_f)}{w_3 + w_4}}{\mathrm{SE}(\hat{\beta}_{m\_meta})} \end{matrix}$$

and correlation matrix:

$$\boldsymbol{\Sigma} = \begin{matrix} 1 & \mathrm{cor}(\hat{\beta}_{f_{meta}}, \hat{\beta}_{m\_meta}) \\ \mathrm{cor}(\hat{\beta}_{f_{meta}}, \hat{\beta}_{m\_meta}) & 1 \end{matrix}$$

where

$$\mathrm{cor}(\hat{\beta}_{f_{meta}}, \hat{\beta}_{m_{meta}}) = \left(\left(\frac{w_1}{w_1+w_2}\right)\left(\frac{2w_3}{w_3+w_4}\right)\mathrm{var}(\hat{b}_f) + \left(\frac{w_4}{w_3+w_4}\right)\left(\frac{2w_2}{w_1+w_2}\right)\mathrm{var}(\hat{b}_m) + \left(\left(\frac{w_1}{w_1+w_2}\right)\left(\frac{w_4}{w_3+w_4}\right) + \left(\frac{2w_2}{w_1+w_2}\right)\left(\frac{2w_3}{w_3+w_4}\right)\right)\mathrm{cov}(\hat{b}_m, \hat{b}_f)\right) / (\mathrm{SE}(\hat{\beta}_{f\_meta})\mathrm{SE}(\hat{\beta}_{m\_meta}))$$

Power is calculated as the proportion of draws where the absolute fetal *or* maternal effect exceeds the relevant critical value from the univariate standard normal distribution.

Distribution of the maximum chi-square statistic from the fetal and maternal GWASs under the null hypothesis of no association and derivation of the correction for multiple testing

The one-degree-of-freedom strategies investigated in this manuscript involve double the number of statistical tests across the genome as the two-degree-of-freedom strategy. We address this issue by dividing the significance threshold for the one-degree-of-freedom strategies by two (i.e. adjusting the threshold for genome-wide significance from $\alpha = 6.6 \times 10^{-9}$ to $\alpha = 3.3 \times 10^{-9}$). To investigate the appropriateness of this procedure, we simulated the distribution of the maximum chi-square statistic (i.e. from potentially correlated fetal and maternal tests of association) under the null hypothesis of no association (1 million draws) whilst varying the sample overlap (we assume 1000 individuals in the maternal test, and 1000 individuals in the fetal test) and the correlation between maternal and fetal phenotype. We calculate the quantile corresponding to the upper 5%, 1% and 0.1% tail of this maximum chi-square distribution. We then determine what upper tail probability of the one-degree-of-freedom central chi-square distribution this value corresponds to (**Supplementary Table 1**). Our results suggest, that even in the case of 100% overlap and $\rho = 0.5$, a Bonferroni correction is not too conservative.

Supplementary Table 1. Upper tail probabilities of the one-degree-of-freedom central chi-square distribution associated with the corresponding quantile from the maximum chi-square distribution.

	$\alpha = 0.05$	$\alpha = 0.01$	$\alpha = 0.001$
0% overlap, $\rho = 0$	0.0253	0.00500	0.000496
50% overlap, $\rho = 0$	0.0251	0.00503	0.000485
100% overlap, $\rho = 0$	0.0252	0.00505	0.000484
0% overlap, $\rho = 0.5$	0.0253	0.00498	0.000487
50% overlap, $\rho = 0.5$	0.0256	0.00503	0.000501
100% overlap, $\rho = 0.5$	0.0269	0.00525	0.000536

The FinnGen cohort

FinnGen (https://www.finngen.fi/en) launched in 2017, is a public-private research project, combining digital healthcare and genomic data. The project aims to provide novel medically and therapeutically relevant insights into human diseases. FinnGen is a partnership between Finnish biobanks and their background organizations (universities and university hospitals) and international pharmaceutical industry partners and the Finnish biobank cooperative (FINBB). All FinnGen partners are listed here: https://www.finngen.fi/en/partners.

The FinnGen cohort consists of 500,000 Finnish individuals with genotyping data and health register information, including data from the birth register. After exclusions dictated by the Early Growth Genetics (EGG) consortium birth weight meta-analysis plan, we were left with three cohorts with genotyping data available i.e., 55,656 children, 11,953 mothers, and 7,790 fathers. In addition, three sub cohorts for which the gestation age was available as well consisted of 12,853 children, 11,423 mothers, and 2,680 fathers. The QC and imputation were done as described in [4], briefly described below.

Samples were genotyped with Illumina (Illumina) and Affymetrix (Thermo Fisher Scientific) array, and genotypes were called with GenCall/zCall and AxiomGT1 algorithms, respectively, after which legacy data was lifted to genome build GRCh38. Individuals with genetically inferred sex not matching the reported sex in registries, high genotype missingness (>5%) and excess heterozygosity (±4 standard deviations) were removed. Variants with high missingness (>2%), low Hardy–Weinberg equilibrium (P <1 × 10–6) and minor allele count < 3 were removed. Chip-genotyped samples were pre-phased with Eagle [5] version 2.3.5 using default parameters, except the number of conditioning haplotypes was set to 20,000.

Genotype imputation was carried out using the population-specific SiSu v.3 reference panel, consisting of high-coverage WGS data from 3,775 Finnish individuals, and the Beagle 4.1 software [6]. After post-imputation QC, variants with imputation INFO scores of <0.6 or MAF values of <0.0001 were excluded.

GWAS of 55,656 individuals in FinnGen with own birth weight was performed using Regenie [7] version 2.2.4 with sex, genotyping batch and the first ten first principal components as covariates.

Ethics statement for FinnGen:

Study subjects in FinnGen provided informed consent for biobank research, based on the Finnish Biobank Act. Alternatively, separate research cohorts, collected prior the Finnish Biobank Act came into effect (in September 2013) and start of FinnGen (August 2017), were collected based on study-specific consents and later transferred to the Finnish biobanks after approval by Fimea (Finnish Medicines Agency), the National Supervisory Authority for Welfare and Health. Recruitment protocols followed the biobank protocols approved by Fimea. The Coordinating Ethics Committee of the Hospital District of Helsinki and Uusimaa (HUS) statement number for the FinnGen study is Nr HUS/990/2017.

The FinnGen study is approved by Finnish Institute for Health and Welfare (permit numbers: THL/2031/6.02.00/2017, THL/1101/5.05.00/2017, THL/341/6.02.00/2018, THL/2222/6.02.00/2018, THL/283/6.02.00/2019, THL/1721/5.05.00/2019 and THL/1524/5.05.00/2020), Digital and population data service agency (permit numbers: VRK43431/2017-3, VRK/6909/2018-3, VRK/4415/2019-3), the Social Insurance Institution (permit numbers: KELA 58/522/2017, KELA 131/522/2018, KELA 70/522/2019, KELA 98/522/2019, KELA 134/522/2019, KELA 138/522/2019, KELA 2/522/2020, KELA

16/522/2020), Findata permit numbers THL/2364/14.02/2020, THL/4055/14.06.00/2020, THL/3433/14.06.00/2020, THL/4432/14.06/2020, THL/5189/14.06/2020, THL/5894/14.06.00/2020, THL/6619/14.06.00/2020, THL/209/14.06.00/2021, THL/688/14.06.00/2021, THL/1284/14.06.00/2021, THL/1965/14.06.00/2021, THL/5546/14.02.00/2020, THL/2658/14.06.00/2021, THL/4235/14.06.00/2021, Statistics Finland (permit numbers: TK-53-1041-17 and TK/143/07.03.00/2020 (earlier TK-53-90-20) TK/1735/07.03.00/2021, TK/3112/07.03.00/2021) and Finnish Registry for Kidney Diseases permission/extract from the meeting minutes on 4th July 2019.

The Biobank Access Decisions for FinnGen samples and data utilized in FinnGen Data Freeze 11 include: THL Biobank BB2017_55, BB2017_111, BB2018_19, BB_2018_34, BB_2018_67, BB2018_71, BB2019_7, BB2019_8, BB2019_26, BB2020_1, BB2021_65, Finnish Red Cross Blood Service Biobank 7.12.2017, Helsinki Biobank HUS/359/2017, HUS/248/2020, HUS/430/2021 §28, §29, HUS/150/2022 §12, §13, §14, §15, §16, §17, §18, §23, §58, §59, HUS/128/2023 §18, Auria Biobank AB17-5154 and amendment #1 (August 17 2020) and amendments BB_2021-0140, BB_2021-0156 (August 26 2021, Feb 2 2022), BB_2021-0169, BB_2021-0179, BB_2021-0161, AB20-5926 and amendment #1 (April 23 2020) and it´s modifications (Sep 22 2021), BB_2022-0262, BB_2022-0256, Biobank Borealis of Northern Finland_2017_1013, 2021_5010, 2021_5010 Amendment, 2021_5018, 2021_5018 Amendment, 2021_5015, 2021_5015 Amendment, 2021_5015 Amendment_2, 2021_5023, 2021_5023 Amendment, 2021_5023 Amendment_2, 2021_5017, 2021_5017 Amendment, 2022_6001, 2022_6001 Amendment, 2022_6006 Amendment, 2022_6006 Amendment, 2022_6006 Amendment_2, BB22-0067, 2022_0262, 2022_0262 Amendment, Biobank of Eastern Finland 1186/2018 and amendment 22§/2020, 53§/2021, 13§/2022, 14§/2022, 15§/2022, 27§/2022, 28§/2022, 29§/2022, 33§/2022, 35§/2022, 36§/2022, 37§/2022, 39§/2022, 7§/2023, 32§/2023, 33§/2023, 34§/2023, 35§/2023, 36§/2023, 37§/2023, 38§/2023, 39§/2023, 40§/2023, 41§/2023, Finnish Clinical Biobank Tampere MH0004 and amendments (21.02.2020 & 06.10.2020), BB2021-0140 8§/2021, 9§/2021, §9/2022, §10/2022, §12/2022, 13§/2022, §20/2022, §21/2022, §22/2022, §23/2022, 28§/2022, 29§/2022, 30§/2022, 31§/2022, 32§/2022, 38§/2022, 40§/2022, 42§/2022, 1§/2023, Central Finland Biobank 1-2017, BB_2021-0161, BB_2021-0169, BB_2021-0179, BB_2021-0170, BB_2022-0256, BB_2022-0262, BB22-0067, Decision allowing to continue data processing until 31st Aug 2024 for projects: BB_2021-0179, BB22-0067,BB_2022-0262, BB_2021-0170, BB_2021-0164, BB_2021-0161, and BB_2021-0169, and Terveystalo Biobank STB 2018001 and amendment 25th Aug 2020, Finnish Hematological Registry and Clinical Biobank decision 18th June 2021, Arctic biobank P0844: ARC_2021_1001.

R code used to perform empirical analyses of birth weight

```
#Input files: maternal GWAS summary results statistics of fetal birth weight (fetal_GWAS) and
maternal birth weight (maternal_GWAS) downloaded from
https://www.decode.com/summarydata/
#Each of the two GWAS summary results statistics is formatted into below 9 columns:
#CHR: chromosome number
#BP: base pair position
#EA: effect allele
#NEA: non-effect allele
#EAF: effect allele frequency
#Beta: beta estimate from a GWAS
#SE: standard error of beta
#P: p-value
#N: ample size

# Read in files
fetal <- read.table(fetal_GWAS, header=T)
maternal <- read.table(maternal_GWAS, header=T)

#Rename columns
colnames(fetal) <- c("CHR", "SNP","BP", "ea_fetal", "nea_fetal", "eaf_fetal", "Beta_fetal",
"SE_fetal","p_fetal","n_fetal")
colnames(maternal) <- c("CHR", “SNP", "BP", "ea_maternal", "nea_maternal", "eaf_maternal",
"Beta_maternal", "SE_maternal", "p_maternal", "n_maternal")

#Merge files
data <- merge(fetal, maternal, by=c("SNP","CHR","BP"))

#Check the effect alleles are the same
#If alleles do not match, switch maternal alleles and change the sign of the beta coefficient
data$Beta_maternal_new <- ifelse(data$ea_fetal == data$nea_maternal, data$Beta_maternal*(-1),
data$Beta_maternal)
data$ea_maternal_new <- ifelse(data$ea_fetal == data$nea_maternal, data$nea_maternal ,
data$ea_maternal)
data$nea_maternal_new <- ifelse(data$ea_fetal == data$nea_maternal, data$ea_maternal ,
data$nea_maternal)
data$eaf_maternal_new <- ifelse(data$ea_fetal == data$nea_maternal, 1-data$eaf_maternal ,
data$eaf_maternal)

data <- subset(data, data$ea_fetal == data$ea_maternal_new & data$nea_fetal ==
data$nea_maternal_new)

data$Beta_maternal <- data$Beta_maternal_new
data$ea_maternal <- data$ea_maternal_new
data$nea_maternal <- data$nea_maternal_new
data$eaf_maternal <- data$eaf_maternal_new

#Read in the intercept of bivariate LD score regression of fetal GWAS and maternal GWAS
int <- 0.1716
```

```
#Calculate the direct fetal and maternal effects, their variances, standard errors and p-values, and
their covariance
data$fetal_beta_adjusted <- ((4/3)*data$Beta_fetal) - ((2/3)*data$Beta_maternal)
data$maternal_beta_adjusted <- ((4/3)*data$Beta_maternal) - ((2/3)*data$Beta_fetal)

data$fetal_var_adjusted <- ((16/9)*(data$SE_fetal)^2) + ((4/9)*(data$SE_maternal)^2) -
((16/9)*int*data$SE_fetal*data$SE_maternal)
data$fetal_se_adjusted <- sqrt(data$fetal_var_adjusted)
chisq_fetal_adj <- (data$fetal_beta_adjusted^2)/(data$fetal_var_adjusted)
data$pval_fetal_adj <- pchisq(chisq_fetal_adj, df = 1, ncp = 0, lower.tail = FALSE, log.p = FALSE)

data$maternal_var_adjusted <- ((16/9)*(data$SE_maternal)^2) + ((4/9)*(data$SE_fetal)^2) -
((16/9)*int*data$SE_fetal*data$SE_maternal)
data$maternal_se_adjusted <- sqrt(data$maternal_var_adjusted)
chisq_maternal_adj <- (data$maternal_beta_adjusted^2)/(data$maternal_var_adjusted)
data$pval_maternal_adj <- pchisq(chisq_maternal_adj, df = 1, ncp = 0, lower.tail = FALSE, log.p =
FALSE)

data$covar <- ((20/9)*int*data$SE_fetal*data$SE_maternal) - ((8/9)*(data$SE_fetal)^2)-
((8/9)*(data$SE_maternal)^2)

#Perform two-degree-of-freedom test
effects <- matrix(nrow = 1, ncol = 2)
sigma <- matrix(nrow = 2, ncol = 2)
chisq_2df <- vector(length= nrow(data))
singular_matrix <- vector(length= nrow(data), mode = "integer")

for (i in 1:nrow(data)) {
  if(i%%1000000==0){print(i)}
  tmp <- try({
    effects <- matrix(c(data$fetal_beta_adjusted[i], data$maternal_beta_adjusted[i]), nrow=1, ncol=2,
byrow=TRUE)
    sigma <- matrix(c(data$fetal_var_adjusted[i], data$covar[i], data$covar[i],
data$maternal_var_adjusted[i]), nrow=2, ncol=2, byrow=TRUE)
    chisq_2df[i] <- effects%*%solve(sigma) %*%t(effects)
  }, silent = T)
  if("try-error" %in% class(tmp)){singular_matrix[i] <- 1}
}

data$singular_matrix <- singular_matrix
data$pval_2df <- pchisq(chisq_2df, df = 2, ncp = 0, lower.tail = FALSE, log.p = FALSE)

#Perform one-degree-of-freedom meta-analysis
#Perform meta-analysis of fetal effect
data$b_f1 <- data$Beta_fetal
data$b_f2 <- 2*data$Beta_maternal

data$var_bm <- data$SE_maternal^2
data$var_b_f2 <- 4*data$var_bm
data$var_b_f1 <- data$SE_fetal^2
```

```
data$w1 <- data$var_b_f1
data$w2 <- data$var_b_f2

#Perform inverse variance weighted meta-analysis of fetal effect estimates
data$beta_meta_fetal <- (data$b_f1/data$w1 + data$b_f2/data$w2)/(1/data$w1 + 1/data$w2)
data$cov_b_f1_b_f2 <- int*sqrt(data$var_b_f1)*sqrt(data$var_b_f2)

#Calculate variance of beta_meta
data$var_beta_meta_fetal <- 1/((1/data$w1)+(1/data$w2)) +
2*data$cov_b_f1_b_f2*((1/data$w1)/(1/data$w1+1/data$w2))*((1/data$w2)/(1/data$w1+1/data$
w2))

data$chisq_fetal <- (data$beta_meta_fetal)^2/data$var_beta_meta_fetal
data$p_meta_fetal <- pchisq(data$chisq_fetal, df = 1, ncp = 0, lower.tail = FALSE, log.p = FALSE)

#Perform meta-analysis of maternal effect
data$b_m1 <- data$Beta_maternal
data$b_m2 <- 2*data$Beta_fetal

data$var_bf <- data$SE_fetal^2

data$var_b_m2 <- 4*data$var_bf
data$var_b_m1 <- data$SE_maternal^2

data$w1 <- data$var_b_m1
data$w2 <- data$var_b_m2

#Perform inverse variance weighted meta-analysis of maternal effect estimates
data$beta_meta_maternal <- (data$b_m1/data$w1 + data$b_m2/data$w2)/(1/data$w1 +
1/data$w2)

data$cov_b_m1_b_m2 <- int*sqrt(data$var_b_m1)*sqrt(data$var_b_m2)

#Calculate variance of beta_meta
data$var_beta_meta_maternal <- 1/((1/data$w1)+(1/data$w2)) +
2*data$cov_b_m1_b_m2*((1/data$w1)/(1/data$w1+1/data$w2))*((1/data$w2)/(1/data$w1+1/dat
a$w2))

data$chisq_maternal <- (data$beta_meta_maternal)^2/data$var_beta_meta_maternal
data$p_meta_maternal <- pchisq(data$chisq_maternal, df = 1, ncp = 0, lower.tail = FALSE, log.p =
FALSE)

#Select below variables to be reported:
#ea_fetal: effect allele
#nea_fetal: non-effect allele
#Beta_fetal: beta estimate from the fetal GWAS
#SE_fetal: standard error of beta_fetal from the fetal GWAS
#p_fetal: p-value from the fetal GWAS
#n_fetal: sample size from the fetal GWAS
#Beta_maternal: beta estimate from the maternal GWAS
#SE_maternal: standard error of beta_maternal from the maternal GWAS
```

```
#p_maternal: p-value from the maternal GWAS
#n_maternal: sample size from the maternal GWAS
#fetal_beta_adjusted: direct fetal effect
#fetal_se_adjusted: standard error of fetal_beta_adjusted
#pval_fetal_adj: p-value of fetal_beta_adjusted
#maternal_beta_adjusted: direct maternal effect
#maternal_se_adjusted: standard error of maternal_beta_adjusted
#pval_maternal_adj: p-value of maternal_beta_adjusted
#pval_2df: p-value from the two-degree-of-freedom-test
#p_meta_fetal: p-value from the one-degree-of-freedom meta-analysis of fetal effect
#p_meta_maternal: p-value from the one-degree-of-freedom meta-analysis of maternal effect

data <- data[,c("CHR", "SNP", "BP", "ea_fetal", "nea_fetal",
                "Beta_fetal", ”SE_fetal", "p_fetal", "n_fetal",
                "Beta_maternal", "SE_maternal", "p_maternal", "n_maternal",
                "fetal_beta_adjusted", "fetal_se_adjusted", "pval_fetal_adj",
                "maternal_beta_adjusted", "maternal_se_adjusted", "pval_maternal_adj",
                "pval_2df", "p_meta_fetal", "p_meta_maternal")]

write.table(data, file=”dingo_results.txt”, quote=F, col.names=T, row.names=F)
```

Supplementary References

1. Manichaikul, A. *et al.* Robust relationship inference in genome-wide association studies. *Bioinformatics* **26**, 2867-73 (2010).
2. Warrington, N.M. *et al.* Maternal and fetal genetic effects on birth weight and their relevance to cardio-metabolic risk factors. *Nat Genet* **51**, 804-814 (2019).
3. Jiang, L. *et al.* A resource-efficient tool for mixed model association analysis of large-scale data. *Nat Genet* **51**, 1749-1755 (2019).
4. Kurki, M.I. *et al.* FinnGen provides genetic insights from a well-phenotyped isolated population. *Nature* **613**, 508-518 (2023).
5. Loh, P.R. *et al.* Reference-based phasing using the Haplotype Reference Consortium panel. *Nat Genet* **48**, 1443-1448 (2016).
6. Browning, B.L. & Browning, S.R. Genotype Imputation with Millions of Reference Samples. *Am J Hum Genet* **98**, 116-26 (2016).
7. Mbatchou, J. *et al.* Computationally efficient whole-genome regression for quantitative and binary traits. *Nat Genet* **53**, 1097-1103 (2021).
