# Supplementary material for: DINGO: increasing the power of locus discovery in maternal and fetal genome-wide association studies of perinatal traits
Source: Nat Commun. 2024 Oct 26;15:9255. doi: 10.1038/s41467-024-53495-9 (PMC11513127; doi:10.1038/s41467-024-53495-9)
Supplement: Supplementary file 2 — Description of Additional Supplementary Files [file 41467_2024_53495_MOESM2_ESM.pdf]

Description of Additional Supplementary Files

File Name: Supplementary Data 1

Description: Comparison of power and type I error rate for locus discovery among one degree of freedom test, one degree of freedom meta-analysis and two degree of freedom DINGO test using asymptotic calculation and simulation.

File Name: Supplementary Data 2

Description: Genetic variants associated with birth weight at genome-wide significance identified in the two degree of freedom test.

File Name: Supplementary Data 3

Description: Genetic variants associated with own or offspring birth weight identified at genome-wide levels of significance but not in the two degree of freedom test.

File Name: Supplementary Data 4

Description: Genetic variants associated with birth weight identified in MTAG but not in separate GWAS of own or offspring birth weight.

File Name: Supplementary Data 5

Description: Genetic variants associated with birth weight identified in one degree of freedom meta-analyses.

File Name: Supplementary Data 6

Description: Replication of 8 SNPs from the 2 df DINGO test in the FinnGen cohort

File Name: Supplementary Data 7

Description: Phenome-wide association analysis for the novel genetic variants associated with birth weight identified in the DINGO one degree of freedom meta-analysis (n = 68) using FUMA and GWAS Catalog. FUMA searches for all genetic variants in linkage disquilibrium (user-defined r^2) in the GWAS Catalog and reports the strongest association.

File Name: Supplementary Data 8

Description: Replication of 68 SNPs from the one-degree-of-freedom meta-analysis in the FinnGen cohort

File Name: Supplementary Data 9

Description: Comparison of expected and observed significant associations between 10,000,000 genetic variants and permuted own (n = 312,882) and offspring birth weight (n = 312,882) in the UK Biobank. 10,000 permutations were performed using a thinned set of one SNP every 10,000 variants (i.e. 1000 independent SNPs) yielding 10^7 statistical tests overall.
